# Spatio-Thermal Variability and Behaviour as Bio-Thermal Indicators of Heat Stress in Dairy Cows in a Compost Barn: A Case Study

**DOI:** 10.3390/ani11051197

**Published:** 2021-04-21

**Authors:** Frederico Márcio Corrêa Vieira, Allessandro Augusto Soares, Piotr Herbut, Edgar de Souza Vismara, Dorota Godyń, Aline Cristina Zambiasi dos Santos, Tainara da Silva Lambertes, Wellington Felipe Caetano

**Affiliations:** 1 Biometeorology Study Group and Federal University of Technology—Paraná (UTFPR), Dois Vizinhos 85660-000, Brazil; allessandrozootecnista@hotmail.com (A.A.S.); p.herbut@ur.krakow.pl (P.H.); edgarvismara@utfpr.edu.br (E.d.S.V.); alinezooutf@hotmail.com (A.C.Z.d.S.); tainaraslambertes@gmail.com (T.d.S.L.); felipewfc2011@hotmail.com (W.F.C.); 2 Department of Rural Building, Faculty of Environmental Engineering and Land Surveying, University of Agriculture in Krakow, 31-120 Kraków, Poland; 3 Department of Production System and Environment, National Research Institute of Animal Production, 32-083 Kraków, Poland; dgodyn80@gmail.com

**Keywords:** crossbred cows, thermal stress, geostatistics, biometeorology

## Abstract

**Simple Summary:**

The thermal distribution inside a compost-bedded pack barn and the behavioural aspects plays an important role in terms of welfare and sustainability for dairy cows. Through a spatial variability assessment of thermal conditions in a compost barn, we found different regions with comfortable or stressful conditions based on air and bed temperature, as well as wind speed. Regarding the behaviour of cows with different number of lactations, we observed a higher probability of water intake in primiparous cows and increased walking behaviour in multiparous cows during the hottest periods. We suggest that special attention must be given to environmental control in a compost barn, mainly during hot seasons, to avoid different hot spots inside the facility. Additionally, with unbalanced environmental resources, the hierarchy of multiparous over primiparous cows might predominate the alleviation of the herd’s thermal stress.

**Abstract:**

The spatial variability and behavioural aspects of compost-bedded pack barns remain unknown in subtropical regions. In this study, we investigated whether spatial variability occurs in the thermal environment of a compost barn and how the behaviour of dairy cows with different numbers of lactations differs in this system. The spatial sampling design comprised a grid of 108 locations inside the facility. At each location, we measured air temperature, relative humidity, wind speed, and bed temperature at 9:00, 12:00, and 15:00. We performed 24-h behavioural observations. Regarding spatial variability, the north face showed high air temperature values, and the distribution of relative humidity varied from the north to the south face. Kriging maps revealed a high bedding temperature trend, indicating heterogeneous ventilation management. Primiparous cows visited the water trough during the hottest hours of the day, whereas multiparous cows displayed a higher probability of walking during these periods. In conclusion, we observed a heterogeneous management of ventilation through the spatial distribution of the thermal environment inside the compost-bedded pack barn, with multiparous cows exhibiting dominance over primiparous cows seeking environmental resources.

## 1. Introduction

Compost-bedded pack barn systems are widespread in subtropical climates. This phenomenon is due to group housing characteristics, which aids better interaction among animals, a reduction in mastitis and lameness caused by the use of bedding, and better thermal control [1,2,3]. However, the thermal environment could become a critical aspect in this system, mainly regarding ventilation management inside the facility. In general, farmers cannot control air temperature and relative humidity but can regulate wind speed ventilation, including natural and mechanical ventilation [4]. Mechanical ventilation has a dual purpose: to provide thermal comfort for cows by increasing convection from their bodies and assisting in bed drying [5]. However, the same authors warned that the homogeneity of thermal and ventilation conditions in the barn is fundamental because cows can cluster in specific areas with higher wind speed, producing substantial heterogeneity in the deposition of faeces and urine inside the barn.

Previous studies on compost-bedded pack barn systems have used the approach related to spatial variability of the thermal environment. A study based on THI and specific enthalpy indices showed that ventilator operation with a range of up to 17 m during summer could not prevent high spatial heterogeneity in this type of housing [6]. The most homogeneous condition of the thermal environment in a compost barn system was under high-volume and low-rotation ventilation, which provided thermal comfort in more than 50% of the barn area [7]. Ensuring uniform conditions throughout the barn is important because thermal stress in a heterogeneous environment can cause disputes in the herd for environmental resources, such as fans, space per cow, and water [8]. Additionally, the hierarchy and dominance of cows in the herd can provide a choice of sites with lower thermal loads, resulting in weaker animals with a higher level of thermal stress than others [9,10].

In homoeothermic animals, heat production resulting from metabolic processes must be exchanged within a body (across cellular and vascular membranes) and between the body and its environment [11]. This phenomenon is a function of macro- and microclimatic factors, their duration and intensity, the surroundings in which they occur, and the biological characteristics of the animals [12]. Considering the high thermal load in the animal environment, thermoregulatory behaviour is a prime indicator of heat stress. 

A reduction in lying time of dairy cows was verified (from 11.3 to 9.4 h d^−1^) by a temperature and humidity index (THI) above 68 in a free-stall environment [13]. Rumination time was also affected by the thermal environment, with a proportional reduction from the THI threshold of 52 [14]. Although previous studies have evaluated the variability in the thermal environment of dairy cows in confinement, with emphasis on the compost barn system, few studies have evaluated these environments in relation to animal behaviour. 

In this study, we investigated the spatial variability of the thermal environment in a compost barn and the behaviour of dairy cows with different numbers of lactations in a subtropical climate.

## 2. Materials and Methods

The experimental procedures were approved by the Ethics Committee on the Use of Animals of the Federal University of Technology-Paraná (CEUA-UTFPR), Paraná, Brazil (protocol no. 2017/015).

### 2.1. Study Location

A trial was conducted between October 2017 and February 2018 at a commercial farm in the southwestern region of Paraná State, Brazil (25°42′31″ S and 53°03′27″ W at 545 m above sea level). The Köppen classification of the region is humid subtropical (mesothermic or Cfa), with average temperatures of −3–18 °C during cold months and temperatures above 22 °C during hot months [15]. The external meteorological data outside the compost barn are presented in Table 1. 

### 2.2. Environmental and Management Features

The compost barn was built in May 2017 with a north/south orientation. The barn was 24.4 m wide, 31.4 m long, and 8 m high to the skylight. Around the barn it had a knee wall of 0.8 m height. However, this structure did not affect the air movement at cow height. It had a roof height of 4.5 m, with open sides and wood-shaving beds. The ridge venting material had an aluzinc cover, with 48 cm wide opening, along at the eaves (1,5 long). The compost barn had an aluzinc cover without Styrofoam, and the feeding area and water troughs were external. Thus, the barn is partially blocked on west side with building wing. The ventilation system consisted of eight fans (DeLaval brand, model DF 1250; DeLaval, Botkyrka, Sweden), with low-volume and high-speed (LVHS), a 1 hp engine, airflow capacity of 34,000 m^3^ h^−1^, and propeller diameter of 1250 mm. The distance between fan rows was 10 m and between fans in a same row was 3.60 m. The ventilation system was controlled manually. These fans were triggered during the hottest periods of day, mainly between 12:00 and 15:00, or when the air temperature (based on a dry-bulb thermometer near the fan panel) was above 21 °C. The fans were installed at 3.5 m bed height with a tilt angle of 30°. Thus, a fan was installed pointing to the projection at the base of the next fan (North–South direction) and was capable of moving the air mass at 3 m s^−1^. However, this value is only estimated at the centreline a specified distance (by standard) and is not overall speed as affected at cow level. The bedding was stirred twice a day during each milking to a depth of 20 cm using a six-stem cultivator. The herd size varied between 45 and 62 lactating dairy cows and the average bed-stocking rate varied between 12.35 and 17.02 m^2^ animal^−1^. This variation may be explained because animals at the end of lactation were removed from the herd. Further, animals were also inserted into the batch. However, no assessment was carried out until eight days after the new animal was added to the flock. In summary, eight days of assessment were undertaken regarding the microclimatic and bed temperature, aiming to evaluate the spatial variability of these variables, with 10 microclimatic evaluations for thermal comfort index and 10 behavioural evaluations. All evaluations were preferably performed on weekends and according to the availability of the technical team.

The cows were fed at 08:00, 16:00, and 20:00 h. The feeding space allowance was 0.80 m per animal. Food was provided ad libitum, and no feed push-up occurred. The diet was composed of corn silage (35 kg), soybean meal (1 kg), commercial feed with 20% CP (8 kg), mineral salt (1.1%), and pre-dried silage of Tifton 85 (3 kg).

### 2.3. Animal Management

For this study, we used Holstein × Jersey crossbred cows (*n* = 18), housed together, divided into two treatments of nine animals, according to the number of lactations: (1) primiparous cows, with an average weight of 500 kg and average production of 25 kg of milk day^−1^; and (2) multiparous cows with an average weight of 600 kg and average production of 32 kg of milk day^−1^. During the study, these cows remained in the same environment as other lactating cows. To identify the study animals, non-toxic cloth dyes (Acrilex™) were used to mark the individual cows and the corresponding treatment.

### 2.4. Spatial Variability of the Thermal Environment

The sampling design consisted of a grid with 108 locations inside the facility, uniformly distributed in the monitoring area, and spaced 2.5 m between the points. At each position, we simultaneously measured air temperature (°C), relative humidity (%), wind speed ventilation (m s^−1^), and bed temperature (°C) at a depth of 20 cm, at 09:00, 12:00, and 15:00 h. With two data loggers, we measured each one of the 108 points during these time intervals, as described below. Except for bed temperature, all microclimatic variables were measured with height of 2.0 m from the floor. The wind measurement was oriented in one direction, in front of the fan.

The microclimatic variables of the internal environment of the compost barn (air temperature, relative humidity, and dew-point temperature) were measured using a HOBO U12—013 data logger (Onset Computer Corporation, Bourne, MA, USA) with two external channels. This device had a temperature range of -20 to 70 °C and relative humidity from 5 to 95%, with a precision of ±0.35 °C from 0 to 50 °C. For ambient relative humidity, the precision range was ±2.5% from 10% to 90% to a maximum of ±3.5%. Wind speed was measured using a portable digital propeller anemometer (model MS6252A, Mastech, Guangdong Province, China) with a precision of ±3% from 0.40 to 30.0 m s^−1^. For bed temperature, a thermocouple sensor was coupled to a HOBO H21–002 data logger (Onset Computer Corporation). This device was a long-neck probe kind which was inserted into the pack without disturbance and without releasing any heat. The pack was sampled in the same position of the grid during the time interval described above.

### 2.5. THI and Behaviour Recording

We collected air and dew-point temperature data for 24 h, at 5 min intervals, for use in corresponding behaviour analysis to determine the thermal conditions of the animals during the period. Thus, the THI was calculated according to [16]:

(1) THI=Ta+(0.36×Tdp)+41.5

where Ta is the air temperature (°C) and Tdp is the dew-point temperature (°C).

We performed 24-h behavioural observations using 0/1 sampling, and the focal method previously described [17]. Each observation lasted 10 min, with a 30 min interval between each observation. The observations were performed by three trained observers, blinded to treatment, who alternated between each treatment on each new observation day. Behaviours were evaluated using a previously proposed ethogram [8,18], as described in Table 2.

**Figure 1:**
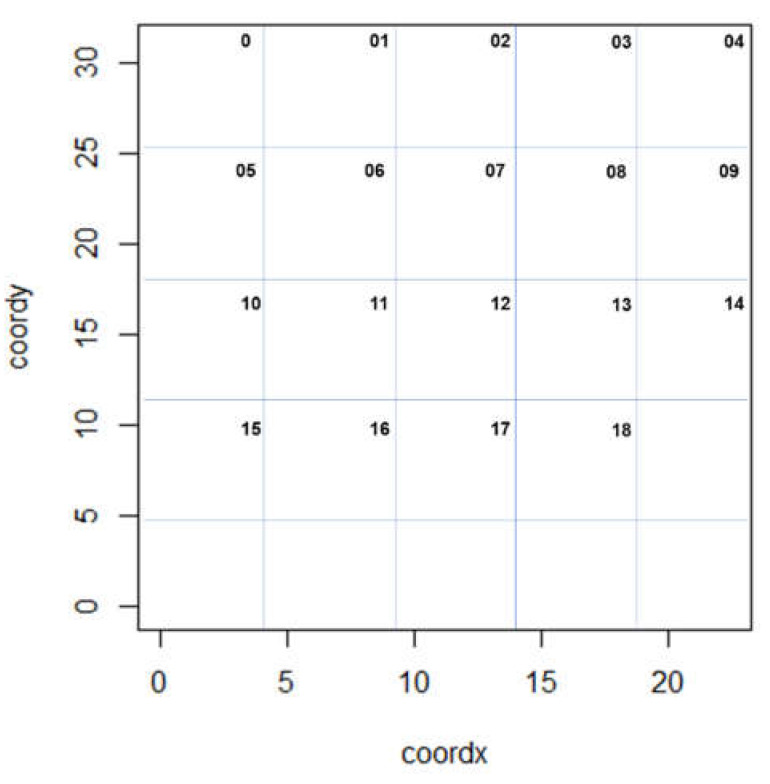
Grid sketch for the positioning animal assessment.

In addition, aiming a better understanding about the manure deposition from congregate in in possible better ventilated areas, we divided the barn in a grid of 18 quadrants uniformed distributed (Figure 1). We registered the positioning of animals (multiparous and primiparous cows) during the behavioural assessment. With this information, a grid map with the positioning frequency was built during the following periods of day: 09:00, 12:00, and 15:00 h.

### 2.6. Statistical Analysis

#### 2.6.1. Geostatistics

Geostatistics, using ordinary kriging, were used to modulate the spatial variability of ambient temperature and relative humidity, as well as wind speed ventilation and bed temperature through a semivariogram fit. The theoretical models were fitted by calculating a previously proposed semivariogram [19].

$$\gamma \left(h\right)=\frac{1}{2N\left(h\right)}\;\overset{N\left(h\right)}{\underset{i=1}{\sum }}{\left[Z\left(xi\right)-Z\;\left(xi+h\right)\right]}^{2}$$

where γ (h) = semivariance and sampling obtained through the achieved results and N(h) = the number of experimental pairs of observations $Z\left(x_{i}\right)$ and $Z\left(x_{i}+h\right)$ separated by a distance h.

To analyse the degree of spatial dependence (DSD), a previously described classification was used [20], where the DSD was classified as strong (DSD ≥ 75%), moderate (25% < DSD < 75%), or weak (DSD ≤ 25%). The semivariogram models considered were the Matérn, cubic, circular, spherical, and Gaussian, which were fitted to the semivariogram using the likelihood method. For geostatistical analysis and construction of kriging maps, R software was used [21], using the geoR package [22].

#### 2.6.2. THI and Behavioural Analysis 

The data related to the THI and behaviour were analysed using Bayesian inference. The primary reason for using this method was the low number of experimental units. The Bayesian method is an appropriate and accurate means of estimation for small samples because of the use of iterative methods, such as the Gibbs sampler. This method facilitates the realisation of *N* simulations using a limited dataset [23]. Additionally, the same author discussed other features of this approach, such as random parameters and fixed data and the inclusion of prior information, which might increase the accuracy of the prediction.

For this modelling approach, it was considered that the variable of interest (Y) followed a Poisson distribution with λ. The model considered for each behaviour within each treatment was as follows:

$$Y_{ki}\;\sim \;Poisson\;\left(\lambda_{i}\right)\;Log\;\left(\lambda_{i}\right)=\alpha +\beta *\;X_{ki}+\pi *X_{ki}^{2}+\rho *\;X_{kI}^{3}+u_{k}+\varepsilon_{kI}$$

α ~ Normal (0, 0.001)

β ~ Normal (0, 0.001)

π ~ Normal (0, 0.001)

ρ ~ Normal (0, 0.001)

where X refers to the period of the day that the behaviour was checked, $u_{k}$ is the random effect of days, and the indices *i* and *K* refer to the animal and day, respectively. For the analyses, the brms package in R software was used [24].

## 3. Results

### 3.1. Spatial Variability

Thermal variables of the compost barn presented spatial dependence, from moderate to strong, except for the air temperature in the third measurement interval (15:00–18:00 h), for which a spatial model was not fitted (Table 3).

The air temperature was adjusted for the circular model in the first (09:00–12:00 h) and second (12:00–15:00 h) measurement interval, with a range of 13.39 and 21.93 m, respectively, indicating greater homogeneity among the samples in the second measurement interval. Air temperature also showed strong spatial dependence (DSD ≥ 75%), with DSD values of 100% for both intervals.

**Figure 2:**
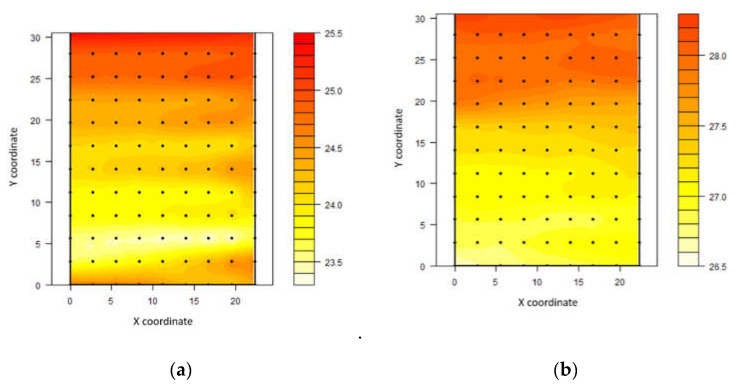
Spatial distribution of air temperature (°C) in compost-bedded pack barn between 09:00 and 12:00 h (**a**) and between 12:00 and 15:00 h (**b**).

Through kriging maps, it was possible to demonstrate that air temperature ranged from 23.3 to 25.5 °C and from 26.5 to 28.3 °C in the first and second measurement intervals, respectively (Figure 2).

The highest air temperature values were recorded on the northern side of the shed. From 09:00 to 12:00 h, the average values in this region oscillated around 25 °C. On the south side at the same time, a narrow range of values, at approximately 24 °C, was recorded. However, the highest heterogeneity was recorded between 12:00 and 15:00 h, with a clear separation between the north and south faces. The thermal amplitude between the two regions was approximately 2 °C. In southern hemisphere the sun is higher on the northern horizon in hot season as compared to northern hemisphere where sun is higher on the southern horizon in warm season. 

Regarding relative humidity, it was observed that the values for this variable were adjusted to the cubic, circular, and Matérn models for the first, second, and third time intervals, respectively. The spatial dependence index values indicated strong spatial dependence for all periods evaluated (DSD ≥ 75%) (Table 3).

The limit of spatial dependence established by the range demonstrated that the first interval presented greater homogeneity among the sampled values, with a range of 45.36 m, followed by the second and third interval, with ranges of 24.41 and 18.08 m, respectively.

**Figure 3:**
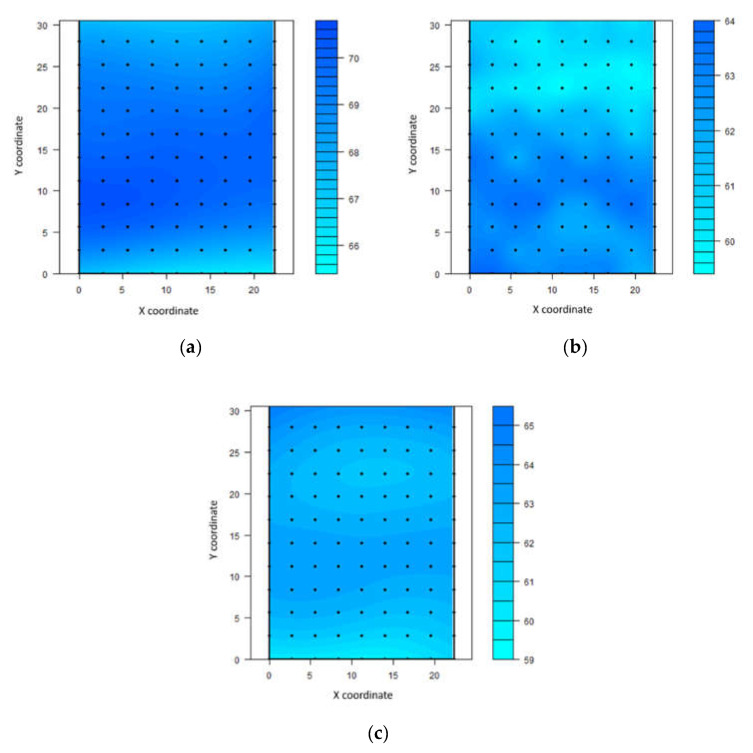
Spatial distribution of relative humidity (%) in compost-bedded pack barn between 09:00 and 12:00 h (**a**), between 12:00 and 15:00 h (**b**), and between 15:00 and 18:00 h (**c**).

On kriging maps, the relative humidity varied in the first interval between 66% and 70%. The second interval ranged from 60% to 64%, and in the third interval, between 59% and 65% (Figure 3).

Except for the first interval, it was observed that the lowest relative humidity values (below 62%) were concentrated near the north face of the shed. A small range of low values was found at the south side between 09:00 and 12:00 h and between 15:00 and 18:00 h. Therefore, the highest concentration of air humidity was located more near the south-central part of the shed.

For wind speed, the models adjusting for this variable were the cubic for the first and third intervals and the Gaussian model for the second interval. The third interval showed a higher spatial dependence range for this variable, followed by the second and first intervals. All three intervals presented moderate spatial dependence (25% < DSD < 75%) (Table 3).

**Figure 4:**
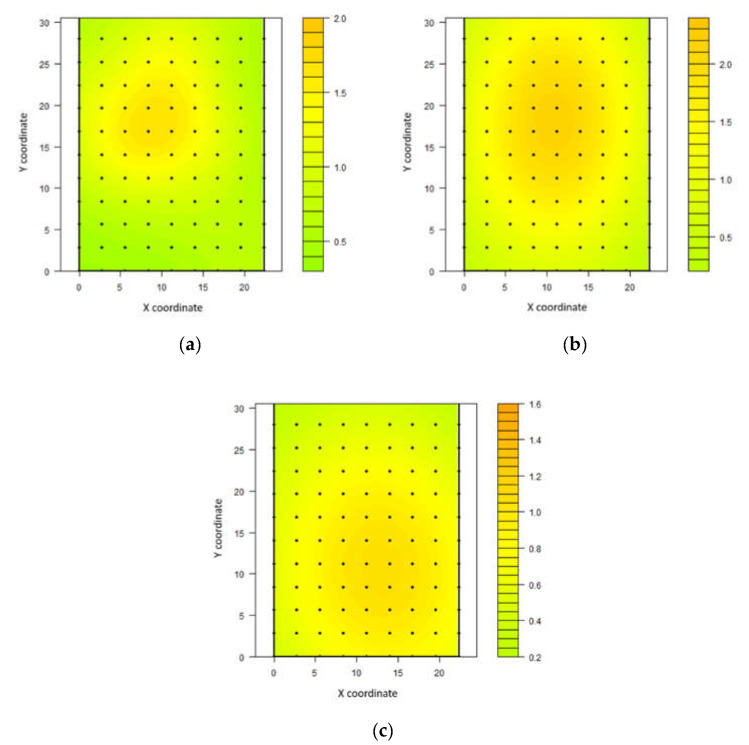
Spatial distribution of wind speed in compost-bedded pack barn (m s^−1^) between 09:00 and 12:00 h (**a**), between 12:00 and 15:00 h (**b**), and between 15:00 and 18:00 h (**c**).

Kriging maps for wind speed (Figure 4) show that the values ranged from 0.3 to 2.0 m s^−1^ in the first interval, from 0.2 to 2.4 m s^−1^ in the second interval, and from 0.2 to 1.6 m s^−1^ in the third measurement interval.

Between 12:00 and 15:00, the ventilation area extended between the north side and the centre of the barn. From 15:00 to 18:00, the core area of greater ventilation was located between the centre and south of the shed. This result showed the heterogeneity of the ventilation in the environment, as well as spatial dependence throughout the day.

The internal temperature of the bed (20 cm depth) exhibited strong spatial dependence. The bed temperature was adjusted using the Matérn model in the first and third intervals and circular for the second interval (Table 3).

The highest range of spatial dependence occurred from 15:00 to 18:00 h, with a value of 24.92 m, followed by 12:00 to 15:00 h, reaching 24.41 m and in the morning, with a value of 18.08 m. In all the intervals evaluated, the bed temperature presented strong spatial dependence (DSD ≥ 75%).

**Figure 5:**
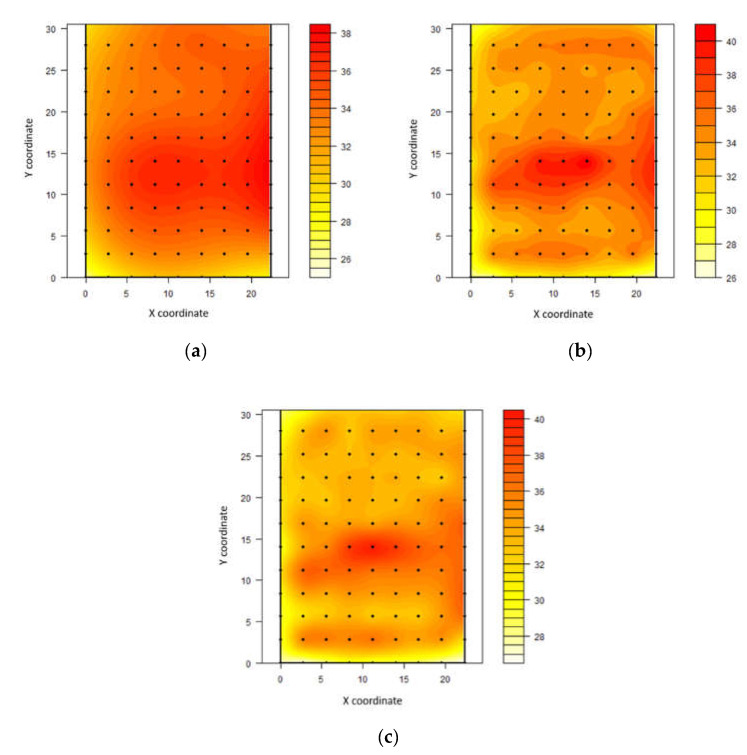
Spatial distribution of bed temperature (°C) in a compost-bedded pack barn between 09:00 and 12:00 h (**a**), between 12:00 and 15:00 h (**b**), and between 15:00 and 18:00 h (**c**).

Through kriging maps, it is possible to visualise the tendency for the highest bed temperatures to occur in areas where the air temperature was low (Figure 5).

During the hottest period, we found a higher bed temperature in the barn centre, which moved south throughout the day. This result corresponds to the observed core of higher wind speed and the lower air temperatures in the barn, indicating a comfortable environment that possibly facilitated a greater concentration of animals in this place. This concentration may have resulted in greater deposition of waste (increasing bed humidity, resulting in a better heat transfer), which caused higher bed temperatures. The bed temperature values ranged from 20.7 to 49.8 °C, representing the high thermal amplitude in these areas and remarkable spatial heterogeneity of the thermal environment. Despite the absence of bed moisture content in this present study, bed temperature at 20 cm is predominantly controlled by bed moisture content. Moreover, the timing of the bed stirring relative to measurements should be noted.

**Figure 6:**
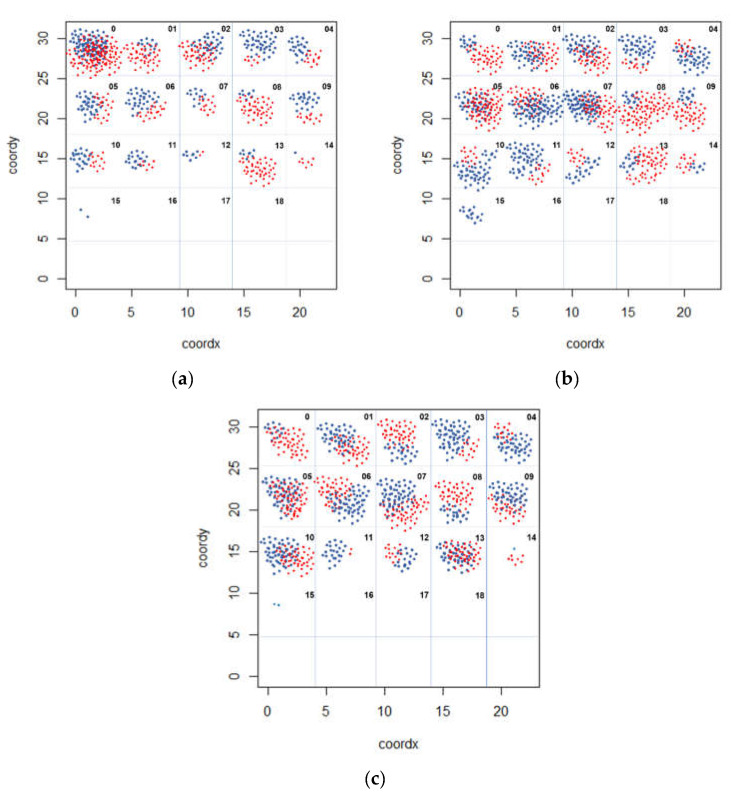
Grid map of the frequency positioning of the animals (primiparous cows—blue dot; multiparous cows—red dot), at 09:00 and 12:00 h (**a**), between 12:00 and 15:00 h (**b**), and between 15:00 and 18:00 h (**c**).

Regarding the positioning of animals throughout the daily periods (Figure 6), we found a high frequency of cows in north side during morning, more concentration in the centre to south side between 12:00 and 15:00 and a centre concentration after 15:00. However, the multiparous cows had positioned in the better ventilated points (Figure 4) mainly during afternoon. This might explain the rising of bed temperature at the same points (Figure 5).

### 3.2. THI and Behaviour 

**Figure 7:**
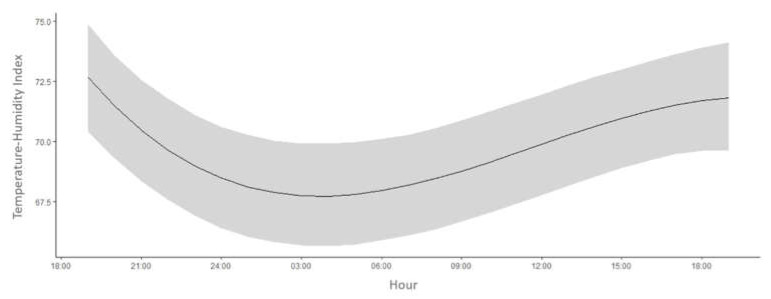
Mean values of Temperature-Humidity Index (THI; 95% Bayesian credibility interval) throughout a 24 h period inside the compost barn.

Higher THI values were observed between 09:00 and 18:00 h and lower values between 24:00 and 06:00 h. The thermal environment of the compost barn presented values lower than 68 between 01:30 and 06:00 h and values between 68 and 75 at other times of the day (Figure 7).

**Figure 8:**
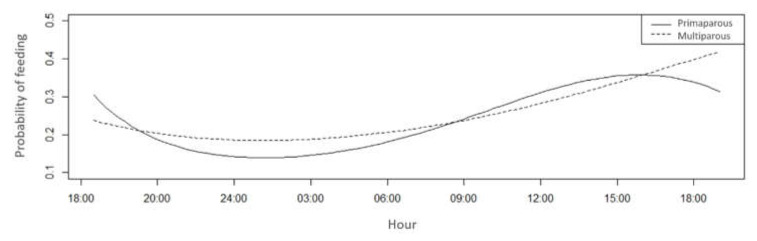
Feeding probability of primiparous (solid line) and multiparous cows (dotted line) in a compost barn.

Based on Bayesian comparisons, the feeding behaviour of primiparous and multiparous cows during the day differed (*p* < 0.05) (Table 4). The pattern of demand for food appeared to have been influenced by milking hours, subsequent feeding, and the thermal conditions of the barn. Multiparous cows were more likely to feed at night and in the early hours of the day, between 8:00 and 20:00 h, whereas primiparous cows were more likely to eat during the day, after 9:00 until approximately 15:00 h (Figure 8).

**Figure 9:**
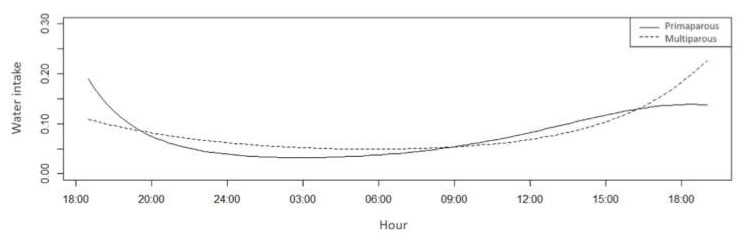
Water intake probability of primiparous (solid line) and multiparous cows (dotted line) in a compost barn.

There was a difference in water intake between treatments during the day (*p* < 0.05) (Table 4). In the present study, water intake was more likely to occur immediately after milking and feed supply, with primiparous cows presenting a higher probability of water intake during the hottest hours of the day and multiparous cows in the cooler hours (Figure 9). In addition to being more likely to consume water at night, multiparous cows were more likely to walk during most hours of the day. 

**Figure 10:**
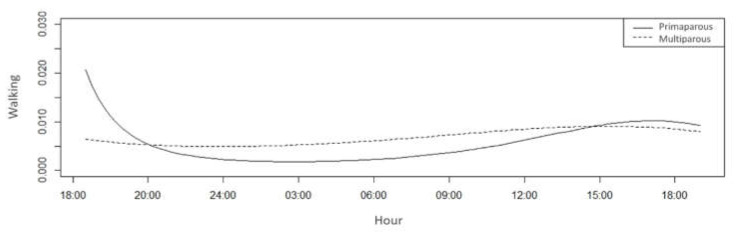
Walking probability of primiparous (solid line) and multiparous cows (dotted line) in a compost barn.

Based on Bayesian comparisons, there was a difference between the treatments in walking behaviour (*p* < 0.05) (Table 4). In the present study, the highest probability of walking for multiparous cows compared with that of primiparous cows was possibly caused by the greater thermal discomfort of these animals, which caused excitation and stress or stimulated the search for areas that facilitated the maintenance of homeostasis (Figure 10).

There was no significant difference in standing rest between treatments during the evaluated period (*p* > 0.05). There was a difference in lying rest between treatments during the day (*p* < 0.05) (Table 4).

**Figure 11:**
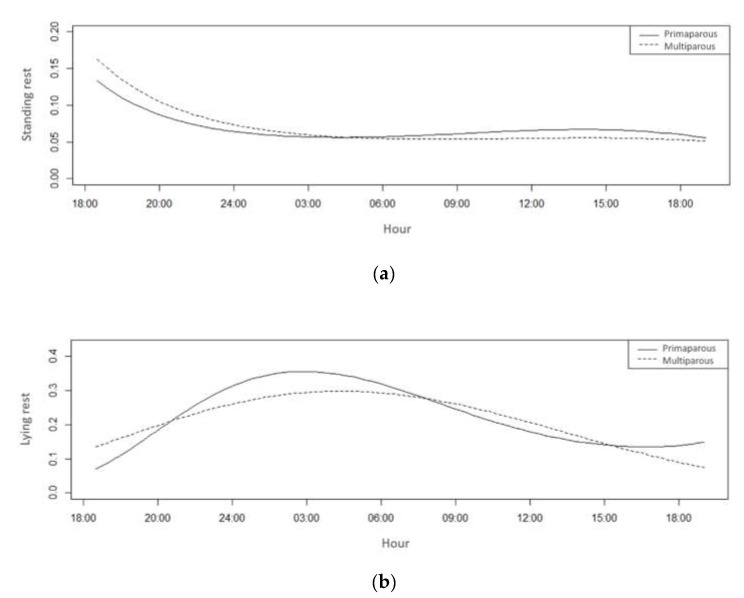
Standing rest (**a**) and lying rest (**b**) probability of primiparous (solid line) and multiparous cows (dotted line) in a compost barn.

The probability of occurrence of both behaviours had a strong association with the variation in THI. Consequently, the multiparous cows presented a higher probability of lying rest at the hottest times (Figure 11).

The highest probability of occurrence for standing rest was between 18:00 and 0:00 h when the THI values presented the highest values, and the highest probability of lying rest occurred between 24:00 and 6:00 h when the index values presented the lowest values.

## 4. Discussion

In this case study, we developed an assessment using a geostatistical approach to verify spatial variability of the thermal environment in a compost-bedded pack barn system. Additionally, we assessed some behavioural responses of dairy cows to verify the effect of the number of lactations on the behaviour of these animals during hot seasons in a subtropical climate. We found heterogeneity in the thermal conditions, mainly temperature (air and bed), but also wind speed. In contrast, we observed some variation in behaviour, which may be associated with environmental stressors. However, towing to the limitation in sample size, we could not infer a strong association between the environment and behaviour. Further, regarding the variation of 45–62 during the study, which varied the pack area per cow of 12–17 m^2^, we stated that this variation did not influenced this study conduction or the results features. Leso et al. (2020) recommended that the suitable pack densities range between 7.4 to above 15 m^2^ per cow. We used an average data from environment and behaviour, and this variation was included in statistical models. However, even with a small sample size, we found significant differences that indicated a possible relationship with thermal conditions, as discussed below. 

### 4.1. Spatial Variability

The optimum temperature zone for dairy cows is between 13 and 18 °C, and the upper critical temperature is between 25 and 28 °C [25]. For relative humidity, the same authors indicated that the ideal range was between 40% and 60%. Beginning at a relative humidity of 78%, the environment is considered to be highly uncomfortable for these animals [26]. Although the relative humidity in the present study ranged between 59 and 70% in the assessed periods, the animals in the present study experienced an environment with critical temperatures between 12:00 h and 18:00 h (above 25 °C), even with ventilation resources (natural and mechanical) in the compost barn. This situation evokes thermoregulatory mechanisms in cows, which, depending on the severity of stress, affect feed intake, feed conversion efficiency, and can decrease milk production efficiency [27,28]. Therefore, based on air temperature alone, we concluded that the animals experienced high thermal discomfort during the afternoon, with greater impairment during the second interval and at the north face of the facility.

The finding that the north face of the barn presented the highest temperatures with lower relative humidity values in two intervals indicates thermal heterogeneity in the facility and, therefore, the inefficiency of the ventilation system to renew air in the barn to achieve high velocities that effectively remove cow heat. Another study sought to evaluate thermal variability in compost barns by comparing natural ventilation and two mechanical ventilation systems (low-volume and high-rotation ventilation and high-volume and low-rotation ventilation) [7]. The authors found values above 26 °C. However, the most critical situation was under natural ventilation, and the regions that experienced temperatures above 29 °C were located at the north side and centre of the barn. In the present study, temperatures were lower (below 25 °C) but heterogeneous. Therefore, this evidence highlights the importance of more precise control of the ventilation system to reduce this spatial variability.

For the three time intervals, the lowest wind speed values occurred on the south side. The tendency of wind speed to be higher in the most central region occurred because the line of action of ventilation system was directed at this region. Additionally, the operation of the fans was performed manually, without any precise criteria on the part of the farmer concerning the thermal environment. These elements contributed to thermal heterogeneity because ventilation was the primary resource for air mass renewal.

The recommended average wind speed for dairy cows in different confined environments is between 1.6 and 3.5 m s^−1^ [29,30,31]. In the present study, the best ventilated area was observed (above 2.0 m s^−1^) in the south-central region during the hot periods. However, the cows’ hair coat layer is the main resistance to heat transfer when the wind speed is above 2 m s^−1^. These results are in agreement with a prior study, where the highest wind speed values of low-volume and high-speed fans (approximately 4.0 m s^−1^) were in the central region of the compost barn, and lower values (approximately 2.5 m s^−1^) were on the sides [32]. As the wind speed increases, there is a decrease in the sensation of heat load, and the positive effects of increased wind speed are more evident under higher air humidity conditions [33]. Thus, the lower wind speed in the periphery could lead to a situation in which animals prefer areas in the central region. At the periphery, the values recorded were below 1.0 m s^−1^, which is outside the recommendations in literature. This condition favours the deposition of waste in a limited space [5]. Because of faeces and urine deposition in a restricted space, this situation may lead to an increase in the bedding temperature in this region.

For effective composting, it is recommended that the bedding temperature be between 43 and 65 °C at a depth of 15 to 31 cm [34]. In the present study, in some points of the facility, there were bedding temperature values far from those recommended for good composting (20.7 to 49.8 °C). Other authors also found bedding temperatures outside the recommended values, such as 33.5 °C [35], 36.1 °C [35], and 31.02 °C [36]. When the recommended temperature is not reached, the compost barn system resembles a semi-composting system in which complete composting does not occur [37].

Kriging maps aided in visualising the trend for the highest values of bedding temperature occurring in areas with low air temperature and where the main ventilation core was formed (Figure 4). The highest bedding temperature found in these places was caused by the greater accumulation of organic matter caused by the higher concentration of animals, wherein they positioned themselves in these areas in search of thermal comfort, closer to the ventilation lines.

Another important aspect regarding bed temperature is the possibility of animals experiencing heat transfer (convection and conduction) at favourable points. In a previous study, the spatial variability of bed temperature in two distinct periods (dry and rainy) revealed high heterogeneity in both situations caused by ventilation management, and revolving bedding occurred [38]. The same authors stated that such situations generate competition and dominance among animals. Accordingly, spaces with higher speed ventilation, lower temperature, and adequate humidity usually have a high concentration of animals searching for an adequate microclimate that favours their thermal exchange with the environment. This heat transfer occurs through conduction (higher proportion of animals lying down) or by convection (a concentration of animals standing in search of the use of ventilation). These results were observed and are discussed in Section 4.2.

### 4.2. THI and Behaviour 

Regarding the THI, the thermal environment of compost barn presented values between 68 and 75 from 6:00 h. Previous studies have indicated that a THI between 68 and 72 represents mild stress in cows, and discomfort conditions occur between 72 and 75 [39,40]. THI values between 68 and 74 may show mild signs of thermal stress, and at a THI greater than 75, the stress results in a drastic decrease in production performance [41]. Therefore, considering the THI within the 24 h cycle, it is evident that the animals presented mild to moderate thermal stress during the experimental period. This stress may have been caused by the spatial variability in the shed caused by ventilation management. 

Regarding feeding behaviour, it should be noted that although feed was provided at the same time (8:00, 16:00, and 20:00 h) in both treatments, primiparous cows were more likely to eat at the hottest times of the day. In contrast, multiparous cows ate during cooler periods, suggesting the dominance of multiparous cows over primiparous cows in access to feed during the cooler times of the day. This hypothesis was reinforced by the social arrangement of cattle. Because they are gregarious animals, they are organised in a social hierarchy that defines which animals have priority access to resources, such as water and feed [42,43]. Among other factors, access to the top of the social hierarchy is determined by sex, territoriality, presence of horns, temperament, sex hormone [44], weight, age [45], breed, and temperament [46]. However, at the feeding trough, hierarchical dominance becomes more prevalent [47].

Considering age and weight, it should be noted that multiparous cows had a preference for feed competition during milder hours than did primiparous cows. Thus, competition regarding the trough line is possible, even when there are no limitations in feed and space availability [48]. In these cases, as shown in the present study, the dominant cows have preferential access to feed and feed at times of higher thermal comfort. The behaviour of ingesting water supports this statement.

Primiparous cows were more likely to drink water during the hottest hours of the day, whereas multiparous cows drank at cooler times. Increasing water intake tends to raise feed intake and thus becomes a priority for cows under stress, along with ventilation resources [8]. The increase in water intake observed at night was to mitigate the damage caused by thermal stress [49]. This explains the results of this research, elucidating the higher probability of multiparous cows consuming water at night. These cows have higher thermal storage than primiparous cows because of the physiological structure derived from the lactation order. 

Another behavioural indicator of thermal stress is walking activity. Pilatti et al. [8] found a difference in the walking duration of primiparous and multiparous cows. Primiparous individuals showed a higher probability of walking during the evaluation period. The authors attributed this to the higher frequency of the number of activities performed by primiparous cows, and it also suggested that this group of animals presents more agitated behaviour than multiparous cows. In turn, we observed the highest level of activity in multiparous females. Walking behaviour reflects the animal’s need to increase its body surface to reduce the thermal load. The elevation of THI was associated with an increase in the number of steps taken by the animals [18]. The same authors stated that the cows took 71.6 steps per hour when the THI was below 72 and 120.8 when THI was equal to or greater than 72. This evidence corroborates the results of the present study. Although the THI revealed mild to moderate stress, multiparous cows have more difficulty dissipating heat, and ventilation resources need to be sought to improve their thermal comfort. 

Regarding lying rest behaviour, we found that multiparous cows were more likely to manifest this behaviour during the hottest periods. During thermal stress, sensitive heat exchanges are preferred, especially conduction, which provides thermal energy transfer to the floor (if the floor is cooler than the animal’s body). As multiparous cows are more sensitive to thermal stress and are more dominant, they seek areas in the facility with greater ventilation. Lying down is one of the predominant forms of dairy cow behaviour in confined systems [9]. 

In a study in which the purpose was to compare the compost barn with a free-stall design, the cows in the compost barn presented a higher frequency of lying behaviour than did cows in the free-stall housing system [37]. The same authors explained that animals in the compost barn were more comfortable than in other systems. Such comfort is caused by the softness of the bed [2]. In another study, longer lying times in cows were influenced by both factors: the nearness of the fans and median bed temperatures [38]. The results of the present study are similar to previous findings in that the bed was moved twice a day to maintain softness. 

## 5. Conclusions

Marked spatial heterogeneity of microclimatic conditions occurred within the compost barn system regarding air and bedding temperature, as well as wind speed, indicating the inefficiency of ventilation management in the facility. Regarding behaviour, we observed the prevalence of the dominant cows seeking environmental resources, with the primiparous cows presenting a higher probability of water intake at the hottest times and multiparous cows demonstrating a higher probability of feed intake at night, as well as walking and lying rest at the hottest times of the day. Owing to the small sample size, additional studies with more animals are recommended to verify these behavioural responses in a compost-bedded pack barn system in a subtropical climate. 

## Figures and Tables

**Table 1:** Average values (mean ± SD) of the external meteorological variables during the trial.

Months	Air Temperature (°C)	Relative Humidity (%)	Wind Speed (m/s)	Wind Direction (°)
October 2017	21.6 ± 5.0	71 ± 20	3.2 ± 1.9	168 ± 75
November 2017	21.8 ± 4.4	67 ± 22	2.3 ± 1.5	188 ± 83
December 2017	23.7 ± 3.7	77 ± 18	1.8 ± 1.5	172 ± 82
January 2018	22.9 ± 3.2	85 ± 14	1.6 ± 1.5	156 ± 81
February 2018	23.0 ± 3.7	73 ± 18	2.4 ± 1.3	172 ± 75

**Table 2:** Behavioural ethogram for dairy cows.

Behaviour	Description
Standing rest	Standing, without performing any activity (rumination, walking, among others)
Lying rest	Lying down, without performing any activity (e.g., rumination)
Feeding	Ingesting food from feed bins
Walking	Displacement in the rest area or feeding line
Water intake	Ingesting water from drinking fountains

**Table 3:** Geostatistical models and adjusted parameters of experimental semivariograms for air temperature, relative humidity, wind speed, and bed temperature.

Time Interval (h)	Parameters	Air Temperature	Relative Humidity	Wind Speed	Bed Temperature
9:00–12:00	Model	Circular	Cubic	Cubic	Matern
	Nugget effect	0.00	1.2 × 10^7^	0.11	1.7 × 10^6^
	Sill	8.5 × 10^4^	2.0 × 10^8^	0.30	1.7 × 10^7^
	Range (m)	15.39	45.36	30.24	18.08
	DSD (%)	100	94	63	88
	Spatial dependence	Strong	Strong	Moderate	Strong
12:00–15:00	Model	Circular	Circular	Gaussian	Circular
	Nugget effect	0.00	0.00	0.19	0.00
	Sill	6.0 × 10^−4^	2.5 × 10^7^	0.70	2.5 × 10^7^
	Range (m)	21.93	24.41	30.2	24.41
	DSD (%)	100	100	72	100
	Spatial dependence	Strong	Strong	Moderate	Strong
15:00–18:00	Model	Cubic	Matern	Cubic	Matern
	Nugget effect	0.00	1.7 × 10^6^	0.10	10 × 10^6^
	Sill	0.00	1.7 × 10^7^	0.29	1.9 × 10^7^
	Range (m)	0.00	18.08	44.25	24.92
	DSD (%)	0	88	68	95
	Spatial dependence	No adjust	Strong	Moderate	Strong

C0 = Nugget effect; Sill (C0+C1); DSD = Degree of Spatial Dependence (C1/C0 + C1) × 100.

**Table 4:** *A posteriori* estimates of parameters (mean ± standard deviation and credibility intervals) of primiparous and multiparous cow behaviour.

		Percentile		
Parameters	Mean ± SD	2.50%	97.50%	Significance
Feeding				
Δ (α)	0.460 ± 0.022	0.0422	0.889	*
Δ (β)	−0.128 ± 0.033	−0.191	−0.0631	*
Δ (π)	0.0000790 ± 0.0000188	−0.000115	−0.0000413	*
Δ (*ρ)*	0.00614 ± 0.00146	0.00324	0.00894	*
Water intake				
Δ (α)	0.843 ± 0.334	0.195	1.503	*
Δ (β)	−0.210 ± 0.0000291	−0.309	−0.116	*
Δ (π)	−0.000122 ± 0.00225	−0.000182	−0.0000670	*
Δ (*ρ)*	0.00976 ± 0.00225	0.00549	0.0143	*
Walking				
Δ (α)	0.842 ± 0.33	0.195	1.503	*
Δ (β)	−0.210 ± 0.050	−0.309	−0.116	*
Δ (π)	−0.000123 ± 0.000029	−0.000182	−0.0000670	*
Δ (*ρ)*	0.00976 ± 0.0023	0.00549	0.0143	*
Standing rest				
Δ (α)	−0.233 ± 0.300	−0.831	0.349	NS
Δ (β)	−0.0000104 ± 0.0471	−0.0933	0.0904	NS
Δ (π)	−0.0000145 ± 0.0000292	−0.0000723	0.0000415	NS
Δ (*ρ)*	0.000878 ± 0.00217	−0.00329	0.00509	NS
Lying rest				
Δ (α)	−0.902 ± 0.257	−1.410	−0.402	*
Δ (β)	0.182 ± 0.0378	0.109	0.258	*
Δ (π)	0.000109 ± 0.0000217	0.0000663	0.000150	*
Δ (*ρ)*	−0.00840 ± 0.00217	−0.0116	−0.00518	*

Δ (α) = Bayesian comparison between T1 and T2 (intercept); Δ (β) = Bayesian comparison between T1 and T2 (linear effect); Δ (π) = Bayesian comparison between T1 and T2 (quadratic effect); Δ (*ρ) =* Bayesian comparison between T1 and T2 (cubic effect); T1 = primiparous cows; T2 = multiparous cows; NS = not significant; * Statistically different based on Bayesian comparisons (*p* < 0.05).

## Data Availability

Not applicable.
